# Targeting IKKα kinase to prevent tumor progression and therapy resistance

**DOI:** 10.20517/cdr.2019.104

**Published:** 2020-03-21

**Authors:** Carlota Colomer, Irene Pecharroman, Anna Bigas, Lluís Espinosa

**Affiliations:** Cancer Research Program, Institut Mar d’Investigacions Mèdiques, CIBERONC, Hospital del Mar, Barcelona 08003, Spain.

**Keywords:** IKKα, cancer, therapeutic targets, chemoresistance

## Abstract

Cancer therapy has improved considerably in the last years; however, therapeutic resistance is still a major problem that impedes full response to the treatment and the main cause of patient relapse and death. Numerous kinases have been reported to be overactivated in cancer and induce resistance to current therapies. Targeting kinases has proven to be useful for overcoming chemotherapy resistance and thus improving patient outcomes. Inhibitor of kappaB kinase alpha (IKKα) is a serine/threonine kinase that was first described as part of the IKK complex in the nuclear factor-κB (NF-κB) pathway, which regulates several physiological and physiopathological processes such as immunity, inflammation, and cancer. However, the IKKα subunit has been shown to be dispensable for NF-κB activation and responsible of multiple pro-tumorigenic functions. Furthermore, we identified a nuclear active form of IKKα kinase IKKα(p45) that promotes tumor growth and therapy resistance, independent of canonical NF-κB. Improved understanding of resistance mechanisms will facilitate drug discovery and provide new effective therapies. Here, we review the recent publications on the implications of IKKα in cancer initiation, development, and resistance.

## Introduction

Cancer is the second leading cause of death worldwide^[1]^. Specific genetic background, exposure to various environmental stresses, and unhealthy diets are the main factors that lead to the accumulation of molecular changes or mutations that will contribute to the initiation and progression of carcinogenesis.

The mainstream therapeutic approaches to treat cancer patients are surgery, irradiation, and chemotherapy. However, its success is limited due to lack of selectivity for tumor cells over rapidly dividing non-malignant cells (e.g., bone marrow and gastrointestinal tract cells) resulting in several toxic side effects. Tumor response to these highly toxic non-specific chemotherapeutic agents is usually partial, transitory, and unpredictable and very frequently patients develop therapy resistance, which is a leading reason for tumor relapse and poor survival in cancer patients.

In recent years, targeted therapy and immunotherapy has been introduced as a breakthrough for cancer therapy. In contrast to chemotherapy, these therapies are directed against cancer-specific molecules and signaling pathways, which give high specificity toward tumor cells and provides a broader therapeutic window^[2]^. They are also often useful in combination with chemotherapy or radiation to produce additive or synergistic anticancer activity because their toxicity profiles often do not overlap with traditional cytotoxic chemotherapy. However, immunotherapy presents new challenges related with other adverse events, such as cytokine release syndrome and neurological toxicity in the case of chimeric antigen receptor-T cells against CD19 for treatment of B-acute lymphoblastic leukemia.

Deregulation of kinase activity has emerged as a major mechanism by which cancer cells evade normal physiological constraints on growth and survival, making them one of the most intensively pursued classes of drug targets for cancer therapy. To date, 52 kinase inhibitors have received United States Food and Drug Administration approval for cancer treatment^[3]^, and there are considerable efforts to develop selective small molecule inhibitors for a number of other kinases that are implicated in cancer.

One kinase that is increasingly envisioned as a target for the development of novel anti-cancer therapies is IKKα. Traditionally, IKKα has been characterized for its critical role in the regulation of the immune response through the nuclear factor-κB (NF-κB) signaling pathway. More recently, different studies have shown that IKKα is implicated in the pathogenesis and resistance to treatment^[4,5]^ of many human diseases, including cancer^[6]^. Dysregulation of IKKα promotes tumor survival, proliferation, migration, metastasis, and angiogenesis, which are common hallmarks of many human tumors.

## Physiologic IKKα functions

As mentioned above, IKKα is an essential regulator of the NF-κB pathway^[7]^ downstream of multiple proinflammatory factors, such as tumor necrosis factor alpha (TNFα), interleukin-1, and toll-like receptor agonists. In brief, binding of these factors to specific receptors induces the recruitment of TGFβ-activated kinase 1 (TAK1) into the proximity of the IKK complex [formed by the catalytic subunits IKKα and IKKβ and the regulatory subunit NF-κB essential modulator (NEMO)]^[8]^, thereby phosphorylating and activating both kinases in the cytoplasm. A major consequence of IKK activation is the initiation of NF-κB-mediated transcriptional activation of a large subset of genes (more than 150 target genes)^[9,10]^. Although these two kinases have a high degree of structural similarity and are both present in the IKK complex, their downstream substrates and physiologic functions can be quite different. In fact, both IKKα and IKKβ have recently been shown to function independently of each other and to have non-overlapping functions^[11]^.

Several natural compounds and synthetic drugs have shown to inhibit IKK activity and prevent cancer or cell growth in animal models^[12-14]^. However, these compounds are extremely toxic due to its effect on the classical NF-κB pathways, which are indispensable for multiple cellular functions, inflammation, and immunity^[15]^. Importantly, IKKα activity is not required for canonical NF-κB signal activation and regulation of the immune system (that mainly depends on IKKβ), but there is increasing evidence of its involvement in the phosphorylation of NF-κB-unrelated substrates that participate in diverse biological processes, including tumorigenesis^[16]^. A few years ago, our group identified a truncated form of IKKα, generated by the proteolytic cleavage of full length IKKα in the endosomal compartment, with a predicted molecular weight of 45 kD, that we called IKKα(p45) and is present in different cell types but specifically activated in the nucleus of cancer cells^[17]^. The IKKα(p45) form includes the kinase domain but lacks some regulatory domains at the c-terminal region of the protein. We proposed that IKKα(p45) might be responsible for the multiple functions that have been ascribed to nuclear IKKα including histone 3 phosphorylation, regulation of specific gene transcription, cancer progression, and metastasis^[18-23]^. Here, we discuss the latest advances on IKKα and the IKKα(p45) form in cancer initiation, progression, and therapy resistance, as well as the strategies to inhibit its activity.

## IKKα in cancer initiation

One of the first pieces of evidence of an NF-κB-independent role for IKKα in cancer was the observation that IKKα deletion in keratinocytes induces skin squamous cell carcinoma in mice. In particular, it was shown that IKKα binds the chromatin at the 14-3-3σ locus to support its expression, which negatively regulates the cell cycle phosphatase CDC25. In the absence of 14-3-3σ, cells aberrantly proliferate, which results in the loss of skin homeostasis and increased cell transformation^[18]^. Other works also supported and further explored this tumor suppressor function of IKKα in the skin, and suggested an association of the kinase with the transforming growth factor beta (TGFβ) pathway^[24,25]^. It was also shown that mice carrying an IKKα variant that specifically localizes to the nucleus of the keratinocytes develop more aggressive tumors in response to chemical carcinogens^[26]^.

**Figure 1:**
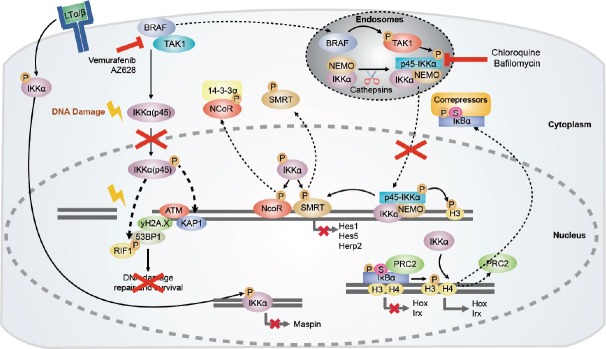
Tumorigenic functions of IKKα and IKKα(p45). In CRC, IKKα phosphorylates the nuclear co-repressors N-CoR and SMRT, inducing their dissociation from chromatin. IKKα(p45) is activated by BRAF and TAK1 in the endosomal compartment, and upon activation phosphorylates histone H3 and SMRT. Inhibitors of the endosomal acidification such as chloroquine or Bafilomycin A1 would prevent IKKα(p45) phosphorylation and impair gene transcription necessary for cancer cell survival. Moreover, nuclear active IKKα contributes to chromatin release of phospho-SUMO-IκBα facilitating its cytoplasmic export. IKKα regulates gene transcription of metastasis repressor Maspin in prostate cancer cells. Under damaging conditions, IKKα(p45) is phosphorylated via BRAF/TAK1/p38-MAPK, inducing its nuclear translocation. In the nucleus, IKKα(p45) induces phosphorylation of ATM and 53BP1, which would favor the recruitment of RIF1 to 53BP1^[4]^. Altogether, the activation of these factors would induce DNA repair and survival of cancer cells. Blocking BRAF activity would block IKKα(p45) activation and cancer cell survival. Arrows indicate activation/regulation/phosphorylation, dashed arrows indicate migration, and the red cross indicates inactivation. CRC: colorectal cancer; IKKα: inhibitor of kappaB kinase alpha; BRAF: serine/threonine-specific protein kinase of the RAF family; TAK1: TGFβ-Activated kinase 1; SMRT: silencing-mediator for retinoid/thyroid hormone receptors; MAPK: mitogen-activated protein kinase; ATM: ataxia telangiectasia mutated gene; NEMO: NF-κB essential modulator; PRC2: polycomb repressive complex 2; KAP1: KRAB-associated protein-1; RIF1: replication timing regulatory factor 1

In contrast, IKKα activity promotes human lung adenocarcinoma in Kirsten Rat Sarcoma (KRAS)-mutant cells and in response to chemical carcinogens. In addition, respiratory epithelial IKKα-deficient mice were strikingly protected from disease. Mechanistically, IKKα potentiates mutant KRAS-induced tumorigenesis in a cell-autonomous fashion, by providing mutant cells with a survival advantage *in vitro* and *in vivo*^[27]^. In a similar setting, it has been shown that nuclear IKKα phosphorylates CREB-binding protein after TNFα stimulation, switching its binding preference from p53 to NF-κB. Hence, IKKα activity not only facilitates NF-κB-dependent gene expression, but also suppresses p53-induced transcription, leading to increased cell proliferation and tumor growth^[28]^ [Figure 1].

In Basal Cell Carcinoma, nuclear IKKα is over-expressed and it directly binds to the *LGR5* promoter, upregulating its expression. By a similar mechanism, murine IKKα contributes to self-renewal of breast cancer progenitors^[29]^. This suggests that IKKα activity can contribute to oncogenic transformation not only through inflammatory-related signals but also through the regulation of stemness-related genes^[30]^. In line with this, our group demonstrated that intestinal-specific deletion of IKKα does not affect intestinal homeostasis but greatly decreases tumor initiation in the *Apc*^*Min/+*^ background, associated with reduced stem cell-related gene transcription and proliferation^[31]^. In a different study, our group identified IKKα as an essential regulator of nuclear Inhibitor of kappaB α, which acts as a switch for multiple developmental- and stemness-related genes such as homeobox (HOX) and iroquois (IRX). IKKα activity on PS-IκBα induces its chromatin release and HOX activation, which is linked to oncogenic keratinocyte transformation^[32]^.

## IKKα in cancer progression

IKKα’s role in cancer is not only limited to its function in cancer initiation but also in cancer progression and metastasis. For instance, in colorectal cancer (CRC) cells, IKKα is aberrantly activated in the nucleus of tumor cells and bound to the promoter of different Notch-dependent genes, including *hes1* and *herp2*^[19]^. Nuclear IKKα phosphorylates the nuclear co-repressor silencing-mediator for retinoid/thyroid hormone receptors (SMRT), leading to its chromatin release^[22]^ and Notch-dependent gene expression^[33]^. General IKK inhibition restores SMRT chromatin binding, thus inhibiting Notch-dependent gene expression and preventing tumor growth in a xenograft model of nude mice. In a similar fashion, IKKα phosphorylates N-CoR, which is closely related to SMRT, in CRC cells, creating a functional 14-3-3 binding domain and facilitating its nuclear export^[20]^. Interestingly, the nuclear active IKKα isoform IKKα(p45) is required to prevent apoptosis in CRC cells, thus increasing tumor growth. Mechanistically, we demonstrated that BRAF is required for the association of active TAK1 with a complex that contains IKKα(p45), full length IKKα, and NEMO, leading to its activation. Importantly, active nuclear IKKα(p45) mediates phosphorylation of SMRT and Histone H3 and is required for BRAF-mediated transformation but has no impact on NF-κB signaling^[34]^.

In prostate cancer cells, nuclear IKKα negatively regulates the expression of *Maspin*, a well-characterized metastasis suppressor, presumably by facilitating the recruitment of DNA methyltransferase activity to its promoter, independent of NF-κB^[35,36]^. Similarly, nuclear active IKKα promotes skin tumorigenesis by regulating *c-Myc*, *Maspin*, and *Integrin-*α6 transcription. Tumors carrying nuclear IKKα mimic the characteristics of human skin tumors with high risk to metastasize^[26]^. These results are in agreement with previous findings of our group indicating that high levels of nuclear active IKKα are predictive of higher metastatic capacity in human squamous cell carcinoma and worse patient outcome^[37]^. In non-small cell lung cancer, IKKα promotes increased cell malignancy through activation of different signaling pathways, depending on its subcellular localization. In particular, cytoplasmic IKKα increases epidermal growth factor receptor (EGFR) and NF-κB activities, whereas nuclear IKKα leads to an increase in c-Myc, P-Smad2/3, and Snail levels^[38]^.

IKKα is also responsible for the tumor-promoting effect of progesterone in breast cancer downstream of receptor activator of NF-κB ligand (RANKL) induction^[39,40]^ and for the metastatic spread of breast cancer cells, which depends on RANKL produced by tumor-infiltrating regulatory T cells^[41]^. IKKα also phosphorylates Estrogen Receptor α, its coactivator AIB1/SRC3, and induces downstream targets such as cyclin D1 and c-myc, promoting breast cancer cell proliferation as well^[42,43]^. In a clinical study, Bennett *et al*.^[44]^ observed that IKKα expression in breast cancer cells is associated with patient outcome independently of their cellular localization. In triple negative breast cancer (TNBC) cells, Notch ligand Jagged1 triggers IKKα-dependent Notch signaling, which is a key pathway regulating TNBC Cancer Stem Cell survival. These data suggest that combination treatments targeting the intersection of the Notch, AKT, and NF-κB pathways have potential therapeutic applications against CSCs in TNBC^[45]^.

## IKKα in therapy resistance

Apart from its role in tumor initiation and progression, recent results from our group demonstrated that IKKα, and, in particular, IKKα(p45), is activated not only in cancer cells carrying mutant BRAF, but also in cells possessing wild-type KRAS and BRAF in response to stimuli that induce DNA damage, such as chemotherapy or irradiation^[4]^. Activation of IKKα(p45) by these damaging agents is BRAF/TAK1/p38-dependent. The most important finding was that damage-induce IKKα(p45) regulates activation of the DNA damage response (DDR) pathway by directly phosphorylating the Ataxia Telangiectasia Mutated (ATM) kinase leading to efficient DNA repair. Consequently, IKKα or BRAF inhibition or downregulation reduces phosphorylation/activation of key DDR elements such as ATM and 53BP1 after exposure of cells to chemo/radiotherapy, leading to sustained DNA damage. We formally demonstrated that IKKα or BRAF inhibition (by precluding DNA damage repair) synergistically enhance the therapeutic potential of the standard of care therapy in CRC (5-FU plus irinotecan), leading to the eradication of chemotherapy-resistant metastatic human tumors *in vivo*. These findings are of paramount significance for the field of cancer therapy since they provide a rationale for using BRAF and IKKα inhibitors as a novel combination strategy for cancer treatment or even for treating patients who have become resistant to single chemotherapy (or radiotherapy) regimes. We are currently evaluating the potential use of EGFR inhibitors, which act upstream of BRAF and therefore IKKα(p45) activation, in combination with DNA damaging agents for the treatment of KRAS and BRAF wild type tumors.

## Strategies to inhibit IKKα

Given the growing evidence that IKKα plays an important role in a number of cancers through direct regulation of multiple elements [Figure 1], the development of selective IKKα inhibitors or molecules that target IKKα (and its downstream pathways) are an attractive approach for the pharmaceutical industry that focuses on cancer therapy. To date, reported inhibitors have either been pan-IKK inhibitors or IKKb selective compounds, which are associated with several side effects that have been extensively reviewed^[12-14]^. Among them, we can find a variety of natural products^[46-48]^, biomolecular and peptide inhibitors^[49]^, and synthetic small-molecule inhibitors^[14,50]^. However, recent efforts have been made to inhibit other elements beyond IKKb. This includes the development of apigenin, which inhibits IKKα and NF-κB/p65 activities and demonstrates to have anti-proliferative and anti-invasive effects in cell-based assays and anticancer efficacy in experimental tumor models^[51]^. Leopizzi *et al*.^[52]^ developed a glucosamine derivative, NCPA [2-(*N*-Carbobenzyloxy)l-phenylalanylamido-2-deoxy-β-d-glucose], which inhibits IKKα nuclear translocation, thus increasing *Maspin* expression and inhibiting cell migration. Another group has reported the first series of selective IKKα inhibitors, which effectively inhibit IKKα-driven p100 phosphorylation in U2OS cells without affecting IKKb-mediated activation of NF-κB^[53]^.

Surprisingly, even though IKK inhibitors have been used for years as reliable tools for research and have proven their positive effects in a variety of experimental cancer models^[34,50,54,55]^, they have not yet been able to move beyond animal studies. As mentioned, this is in part due to the high level of homology between IKKα and IKKb that makes it difficult to discriminate between cancer-related IKK function (several ascribed to IKKα) and NF-κB signaling (IKKb dependent), which is essential for a number of biological processes including inflammation and immunity. Interestingly, Prescott *et al*.^[15]^ and Lee *et al*.^[50]^ described in detail all the commercially available IKKb and IKKα inhibitors, respectively. The recent discovery that BRAF acts upstream of IKKα(p45) phosphorylation and activation in CRC cells, as well as other cancer types, which does not impact on NF-κB activation, might have important implications in this scenario. Consistently, we demonstrated that IKKα(p45) activation can be abolished using different inhibitors of BRAF without affecting classical NF-κB signaling and thus without imposing toxic effects to normal cells. Several of these BRAF inhibitors are currently used in clinical practice for specific anti-cancer therapies including CRC treatment. Another therapeutic option to prevent IKKα(p45) phosphorylation is the use of inhibitors of TAK1, which is the kinase that directly phosphorylates IKKα(p45) in the IKK complex. However, TAK1 participates in the activation of other signaling cascades^[56]^, such as classical NF-κB pathway^[57]^ or TGFβ^[58]^, and in fact there are no TAK1 inhibitors currently approved for treating patients. Because IKKα(p45) activation by TAK1 and BRAF takes place in the endosomal compartment, a third possibility for inhibiting IKKα in CRC cells would be the use of inhibitors of the endosomal vacuolar adenosine triphosphatase (V-ATPase), such as chloroquine or bafilomycin A1. Indeed, Margalef *et al*.^[34]^ demonstrated that treatment of CRC cells with chloroquine or bafilomycin A1 induces apoptosis of cancer cells *in vitro*, and reduces growth and metastasis of a BRAF-mutant xenograft model derived from a patient with acquired resistance to standard chemotherapy.

In human lung adenocarcinoma, IKKα has been shown to be highly expressed and it has been demonstrated that a heat shock protein 90 (HSP90) inhibitor blocks IKK function and has superior efficacy against KRAS-mutant lung adenocarcinoma compared with a specific IKKb inhibitor^[27]^.

Finally, it is important to indicate here that, independently of the tumor type, identifying the mechanisms leading to IKK activation, either cell autonomous (i.e., mutations in specific upstream regulators of the pathway) or induced by adjacent cells from tumor microenvironment, would also allow the identification of new strategies to prevent IKK activation with relevance for cancer therapy.

## Conclusions and perspectives

From the current data, the most realistic possibility for using IKK inhibitors to treat therapy-resistant cancers would involve the combination of compound inhibiting basal or therapy-induced IKKα activity with the more traditional chemo- and radiotherapeutic agents. For instance, our group recently demonstrated that IKKα or BRAF inhibition [leading to abrogation of IKKα(p45) activity] synergizes with chemotherapeutic (DNA-damaging) agents to induce tumor eradication in a xenograft mouse model derived for a chemotherapy-resistant human CRC that had already metastasized to the liver of the patient^[4]^. These findings expand the potential use of combination protocols including IKKα and/or BRAF inhibitors, which are currently used as single agents exclusively in patients carrying BRAF mutated tumors. Because all these experimental approaches have been developed in cells, organoids, xenografts, and mouse models, the possibility of using this kinase inhibitors with one of the multiple immunotherapy strategies that are currently in use or under investigation remains unexplored. These would require the use of humanized mouse models (immune-reconstituted) or novel *in vitro* systems that support the maintenance of mixed populations of cancer and immune cells.

## Declarations

### Copyright

© The Author(s) 2020.
